# Perinatal Factors Associated with Cardiovascular Disease Risk among Preschool-Age Children in the United States: An Analysis of 1999–2008 NHANES Data

**DOI:** 10.1155/2012/157237

**Published:** 2012-05-22

**Authors:** Sarah E. Messiah, Kristopher L. Arheart, Steven E. Lipshultz, Emmalee S. Bandstra, Tracie L. Miller

**Affiliations:** ^1^Division of Pediatric Clinical Research, Department of Pediatrics, University of Miami Leonard M. Miller School of Medicine, Batchelor Children's Research Institute, 580 NW 10th Avenue (D820), Miami, FL 33101, USA; ^2^Department of Epidemiology and Public Health, University of Miami Leonard M. Miller School of Medicine, Miami, FL 33101, USA; ^3^Division of Biostatistics, University of Miami Leonard M. Miller School of Medicine, Miami, FL 33101, USA; ^4^Division of Neonatology, University of Miami Leonard M. Miller School of Medicine, Miami, FL 33101, USA

## Abstract

We examined the relationships between selected perinatal and early infancy factors (maternal smoking during pregnancy, infant low birthweight, breastfeeding, and early introduction of solid foods [<6 months of age] and increased BMI [≥85th, ≥95th percentiles for age, sex]), waist circumference (WC), C-reactive protein (CRP), triglycerides, total cholesterol, low-density lipoprotein (LDL) cholesterol, non-high-density lipoprotein (HDL) cholesterol, and decreased HDL cholesterol during early childhood. The population-based sample included 3,644 3-to-6-year-old Non-Hispanic White (NHW), Hispanic, and Non-Hispanic Black (NHB) children who participated in the 1999–2008 National Health and Nutrition Examination Surveys. Analysis showed that breastfeeding was significantly protective against early childhood obesity (OR 0.43, 95% CI, 0.27–0.69) and the highest quintile for WC (OR 0.58, 95% CI, 0.37–0.32) among NHW, and against the highest quintile of non-HDL cholesterol among NHB (OR 0.56, 95% CI, 0.32–0.98). Additionally, NHW children were significantly more likely to be obese (OR 2.22, 95% CI 1.30–3.78) and have higher CRP levels (OR 1.63, 95% CI, 1.05–2.51) if their mothers smoked during pregnancy. These results support the observation that breastfeeding may be protective against early childhood obesity while maternal smoking during pregnancy is a risk factor for obesity and increased CRP levels among NHW young children.

## 1. Introduction

The theory that events in the perinatal period have latent effects throughout the life course has become increasingly accepted [1]. The literature on the fetal-origins hypothesis strongly suggests that while maternal malnutrition and poor maternal health might not cause major malformations in childhood, such exposures can nevertheless have enduring, or latent, effects such as restricted growth, hypertension, cardiovascular events, and altered renal function in adulthood. [1, 2] These potential maternal environments can be physiological (e.g., maternal metabolic regulation), psychobehavioral (e.g., stress, smoking), and/or ecological (e.g., poverty, unstable food supply).

The literature is emerging on how these environments affect the long-term growth and health of children as they develop into adulthood. [3–5] A recently published review of 135 studies to evaluate factors in early childhood (≤5 years of age) that are the most significant predictors of the development of obesity in adulthood reported that possible early markers of obesity included maternal smoking and maternal weight gain during pregnancy [6]. Probable early markers of obesity included maternal body mass index, childhood growth patterns (early rapid growth and early adiposity rebound), childhood obesity, and father's employment (a proxy measure for socioeconomic status [SES] in many studies). Other recent studies have examined the relationship between prenatal exposures such as smoking during pregnancy and early feeding practices (e.g., breastfeeding, early introduction of solid foods) and adverse cardiovascular health outcomes in preschool age children [7, 8]. Results of these studies showed that maternal smoking during pregnancy was associated with a higher body mass index (BMI) at four years of age in children with a normal birth weight and in those who were small for gestational age at birth [7] and that shorter breastfeeding duration and exclusivity (no formula or solid food) during the first 6 months tended to be associated with increased growth rates for length, weight and BMI between the age of 3 and 6 months but not with the risks of overweight and obesity until the age of 3 years [8]. 

Early childhood is an important stage of growth to examine given that one in four US children under age 5 is either overweight (≥85th to <95th age- and sex-adjusted percentiles for BMI) or obese (≥95th age- and sex-adjusted percentiles for BMI) [9, 10]. Overweight preschool-age children are five times more likely to be overweight during adolescence and more than four times as likely to become obese as adults than are their normal-weight counterparts [11]. Recent studies have shown that obesity in this age range is associated with cardiovascular disease risk factors and varies by ethnic group [12]. The concern is that childhood overweight will contribute to the earlier onset of overall morbidity and mortality in adulthood, making early intervention crucially important [4, 5]. 

Preschool children are at an ideal age to examine these relationships because there are fewer exposures to environmental confounders and interactions compared to older children and adults when it might be much more challenging to distinguish the contribution of the environment versus physiology. The relationship between overweight/obesity, cardiovascular disease (CVD) risk factors and birthweight, mother's smoking status during pregnancy, breastfeeding, and early introduction of solids and how these relationships vary by ethnic group specifically is largely unknown. Therefore, we assessed these relationships in a population-based multiethnic sample of 3-to-6-year-olds to determine the influence of breastfeeding, early introduction of solid foods, smoking during pregnancy, and low birth weight on the prevalence of CVD risk factors.

## 2. Methods

### 2.1. Study Population

The periodic National Health and Nutrition Examination Survey (NHANES) uses a stratified, multistage probability design to capture a representative sample of the civilian, noninstitutionalized US population [13]. This design allows survey results from two or more periods to be combined to increase the sample size and analytic options. Each 2-year period, and any combination of 2-year periods, is a nationally representative sample. To produce estimates with greater statistical reliability for demographic subgroups and rare events, combining two or more 2-year periods of the survey results is strongly recommended [13]. Therefore, for this study, NHANES data files for 1999-2000, 2001-2002, 2003-2004, 2005-2006, and 2007-2008 were combined to form a single analytic file.

### 2.2. Eligibility Criteria

From the above-cited NHANES 1999–2008 data file all Non-Hispanic White (NHW), Hispanic (Mexican American, other Hispanic groups combined), and Non-Hispanic Black (NHB) boys and girls aged 3 to 6 years and their mothers were included. The following measurements were available for analysis in this age group: age, sex, ethnicity, height and weight (for BMI), WC, C-reactive protein (CRP), total cholesterol, high-density lipoprotein (HDL), low-density lipoprotein (LDL) cholesterol, and triglycerides (available in the morning-only randomized subsample).

Using data on response rates found on the CDC website [14], we estimated the total number of 3-to-6-year-old children who were screened in the surveys from 1999–2008 to be 4,627. Of the children screened, 4,091 (88%) participated in the interview and 3,876 (84%) participated in the examination. We eliminated 225 children who were in the “other” ethnic group category (not Hispanic, NHW or NHB) and another 7 children who were diabetic or on metabolic altering drugs; therefore, our analytic sample included 3,644 children (79% of children screened). With a sample size of more than 1000 in each ethnic group (Table 2), it is possible to detect a small effect size of 0.20 at the two-tailed 0.05 level with 80% power [15].

### 2.3. Measures and Data Collection

Persons selected to participate in the NHANES survey were invited to be interviewed in their homes. Household interview data were collected with computer-assisted personal interviewing procedures and included demographic, socioeconomic, dietary, and health-related information. Because the children were so young (less than 7 years old), the mothers answered all questions on their behalf. Mothers were asked questions about their pregnancy with the index child included in this analysis. Specifically included were the responses regarding self-reported smoking status during pregnancy, infant birthweight, whether the infant was breastfed, and if solid foods were introduced in the infant's diet before six months of age. After the interview, each child received a standardized physical examination at a local Medical Examination Center.

Laboratory and anthropometric measurement methods used at the Medical Examination Centers are described in *The NHANES Laboratory/Medical Technologists Procedures Manual*. [16] Briefly, anthropometric measures taken during the standardized examination consisted of barefoot standing height (with a stadiometer), weight with minimal clothing (on a digital, electronic scale) [1], and waist circumference (in the horizontal plane at a point marked just above the right ileum on the midaxillary line, at minimal respiration after normal expiration) [16, 17]. 

Triglycerides and low-density lipoprotein (LDL) cholesterol were measured via blood on a nonfasting subsample (those who were examined in the morning session only) of all children ages 3 to 11 (*N* = 626). All serum blood samples were collected, processed, stored at −20°C, and shipped to the Lipid Laboratory, Johns Hopkins University, Baltimore, MD (lipids) for the1999–2006 surveys and to the University of Minnesota, Minneapolis, MN, for the 2007-2008 survey for analysis [18]. 

High-density lipoprotein (HDL) cholesterol was measured in supernatants after precipitation of apo B-containing lipoproteins with heparin-manganese chloride and removal of excess manganese by precipitation with sodium bicarbonate. Triglycerides were analyzed enzymatically with the use of commercial reagents and was measured in EDTA plasma after hydrolysis by lipoprotein lipase to glycerol and fatty acids. Glycerol is enzymatically phosphorylated and then oxidized to release hydrogen peroxide, which is peroxidized to form a quinine-imine chromophore that can then be read at 490 to 550 nm in a spectrophotometer [18]. Low-density lipoprotein cholesterol (LDL) was derived using the Friedewald calculation (LDL = total cholesterol-HDL cholesterol-triglyceride/5) [19].

CRP was quantified by latex-enhanced nephelometry (CRP present in the test sample forms an antigen antibody complex with the latex particles). Serum blood specimens were processed, stored and shipped to University of Washington, Seattle, WA [18]. Particle-enhanced assays were based on the reaction between a soluble analyte and the corresponding antigen or antibody bound to polystyrene particles. A dilute solution of test sample was mixed with latex particles coated with mouse monoclonal anti-CRP antibodies. Non-HDL cholesterol is defined as total cholesterol minus HDL cholesterol.

### 2.4. Statistical Methods

The highest quintile for CRP, total cholesterol, LDL cholesterol, non-HDL cholesterol and triglycerides and the lowest quintile for HDL cholesterol, for the entire study population were used as cut-points for analysis (Table 1). These cut-points are consistent with the percentages used for abnormal values in studies of older children [20, 21]. Logistic regression analysis was used to explore the relationships between each of the selected perinatal and infant conditions and anthropometric measurements and CVD risk factors for each ethnic group. Child's age and sex, the family's poverty/income ratio, the year of the survey (to account for possible trend over the 10 years of data), and mother's age at the child's birth were included in each model to control for potential confounding effects. Separate analyses were run for the three race/ethnicity categories. Analyses were performed with SAS SURVEY procedures (SAS version 9.2, SAS Institute, Cary, NC) to accommodate the complex sample survey design of the NHANES data. Alpha was set at 0.05 and all tests were two tailed, without correction for multiple comparisons.

## 3. Results

We analyzed data from 3,644 (weighted sample size, 14,535,068) children and their mothers (Tables 2 and 3). Overall, 13.8% of children had a BMI ≥95th percentile and 26.5% had a BMI ≥ 85th percentile for age and sex (Table 1). Approximately 65% of mothers reported breastfeeding while 42% reported introducing solids before 6 months. Almost 10% of the infants were low birth weight (<2500 grams) and 17% of the mothers reported smoking while being pregnant (Table 3).

Table 4 shows that breastfeeding was significantly protective against obesity (BMI ≥ 95th percentile ile for age and sex) (OR 0.43, 95% CI, 0.27–0.69), overweight (OR 0.61, 95% CI, 0.42–0.87), and being in the highest quintile for waist circumference (OR 0.58, 95% CI, 0.37–0.92) among the NHW group and against highest quintile for non-HDL cholesterol among NHB (OR 0.56, 95% CI, 0.32–0.98). Conversely, introducing solid foods before 6 months was not shown to be a risk factor for any CVD risk factors among all ethnic groups.

Smoking during pregnancy was a significant risk factor for obesity (OR 2.22, 95% CI, 1.13–2.83), overweight (OR 1.79, 95% CI 1.13–2.83), and highest quintile of CRP (OR 1.63, 95% CI, 1.05–2.51) among NHW (Table 5).

Table 6 shows that in general low birth weight was not a risk factor for becoming overweight or obese or having elevated CVD risk factors in this age group was associated with a diminished risk among NHW for being overweight (OR 0.33, 95% CI 0.14–0.76) and having the highest quintile of waist circumference (OR 0.34, 95% CI 0.16–0.74).

## 4. Discussion

The results reported here show that CVD risk in young children is associated with perinatal and infancy factors among NHW. Specifically, our analysis shows that breastfeeding is protective against obesity and smoking during pregnancy is a risk factor for obesity in NHW. Low birth weight was not a risk factor for CVD risk factors, particularly among NHW women. With the exception of breastfeeding being protective against the highest quintile of non-HDL cholesterol in NHB, early introduction of solid foods, smoking during pregnancy, and low birth weight were not found to be related to CVD risk factors in early childhood.

Previous NHANES analyses have shown mixed results when analyzing whether or not breastfeeding is protective against children becoming overweight and that there is a dose-dependent effect based on duration. One analysis of infant feeding and child overweight status among 3-to-5-year olds from the NHANES III showed that after adjusting for potential confounders, there was a reduced risk of being overweight for ever-breastfed children compared with those never breastfed [22]. However, there was no reduced risk of being overweight (obese). Furthermore, there was no demonstrable threshold effect or clear dose-dependent effect of the duration of full breastfeeding on being at risk of overweight or overweight (now termed overweight and obese).

Other large population-based studies have also examined whether increasing duration of breastfeeding is associated with a lower risk of overweight in a low-income population of 4-year olds in the United States. Analysis from the Pediatric Nutrition Surveillance System [23] of children up to 60 months of age found that the duration of breastfeeding showed a dose-response, protective relationship with the risk of overweight only among NHW; no significant association was found among NHB or Hispanics, very similar to our results reported here.

Others have examined a broad range of factors that may simultaneously contribute to childhood overweight in a population-based cohort of children followed from birth to 4.5 years, to determine which factors exert the most influence in early life [24]. The Quebec Longitudinal Study of Child Development 1998–2002 (QLSCD) followed a representative sample (*n* = 2103) of children born in 1998 in the Canadian province of Quebec. Measured height and weight were available for 1550 children aged 4.5 years. Results showed that being in the highest quintiles of weight gain between birth and 5 months, as well as maternal smoking during pregnancy, almost doubles the odds of being overweight at 4.5 years.

The Viva La Familia Study was designed to identify genetic and environmental factors affecting obesity and its comorbidities in 1030 Hispanic children from 319 families [25]. Salient independent risk factors for childhood obesity in this cohort of Hispanic children were age, birth weight, maternal obesity, paternal obesity, number of children in the family, and the percentage of awake time spent in sedentary activity. They also reported that breastfeeding might have a small protective effect against childhood obesity, although the authors concluded that residual confounding might exist. The authors reported no significant effect of early introduction of solid foods on childhood obesity, consistent with our findings here. Conversely, a prospective nationally representative cohort study conducted in England, Wales, Scotland, and Northern Ireland [26] included 13,188 singleton children aged 3 years in the Millennium Cohort Study, born between 2000 and 2002, who had complete height/weight data. The main outcome measure was childhood overweight (including obesity) defined by the International Obesity Task Force cut-offs for body mass index. In the fully adjusted model, primarily individual- and family-level factors were associated with early childhood overweight: birthweight z-score, black ethnicity (compared with white), introduction to solid foods <4 months, and smoking during pregnancy. However, in agreement with both the findings here and in the Viva La Familia Study, breastfeeding ≥4 months (compared with none) was associated with a decreased risk of early childhood overweight.

Other population-based studies have shown an association between maternal smoking during pregnancy and childhood obesity. Specifically, a total of 11,653 preschool children participating in the UK Millennium Cohort Study had their weight gain z-scores calculated from 3 to 5 years [27]. In a mutually adjusted model, children were more likely to gain weight rapidly if their mothers smoked during pregnancy. Due to the cross-sectional nature of the current dataset, we were unable to explore longitudinal growth but the concept of catch-up growth has gained recent and increased attention in the literature [28–30]. 

Because our findings are among very young children, they may have implications throughout childhood. Our group has reported previously that risk factors for cardiometabolic disease can be detected as early as the preschool years (12) and 8 years old [31]. Other studies have noted that several CVD risk factors persist strongly and consistently through childhood into adulthood [32, 33]. The Cardiovascular Risk in Young Finns Study was one of the first groups to explore childhood predictors of the metabolic syndrome (MS), a constellation of abnormal waist circumference, insulin resistance, dyslipidemia, and hypertension [32]. In this study, fasting insulin at baseline was related to development of the syndrome after a 6-year follow-up of 1,865 children and adolescents 6-to-18-years-old. Reported results showed that baseline insulin concentration was higher in children who subsequently developed the MS, lending support to the theory that insulin resistance precedes the development of the condition in childhood.

## 5. Study Limitations

In a cross-sectional study, causality cannot be inferred. Blood pressure, insulin and glucose measures, which are important components of metabolic syndrome and risk factors for adult-onset CVD and diabetes, were not collected in this age group and thus were not available for analysis. Because triglycerides and LDL were measured on a subsample of the surveyed children, analyses for these variables may not have sufficient statistical power to detect significant differences. Dietary and physical activity level data were not included because the children in this analysis were so young and their eating and exercise patterns tend to be inconsistent as a result. Smoking status during pregnancy and infant feeding behaviors were self-reported and therefore subject to systematic biases. Finally, genetic influences were not examined.

## 6. Conclusions

This study indicates that behavioral and social factors exert critical influences on the onset of childhood overweight in preschool years among NHW families in particular. Regardless of ethnic background, all women should be advised not to smoke, especially while being pregnant, and should be encouraged to breastfeed, unless contraindicated, as a means of providing optimal nutrition. This may in turn prove to be protective against chronic obesity and later life onset of CVD.

## Acknowledgment

This paper is funded by National Institutes of Health Grant K01 DA 026993 (SEM).

## Figures and Tables

**Table 1:** Values defining cardiovascular disease risk factors for 3-to-6-year-old children in the 1999–2008 NHANES data, by sex and ethnicity.

	Body mass index* kg/m^2^			Waist circumference, cm		C-reactive protein, mg/dL		Total cholesterol, mg/dL		HDL cholesterol, mg/dL		Non-HDL cholesterol, mg/dL		LDL cholesterol, mg/dL		Triglycerides, mg/dL	
Group	*n*	85th %^†^	95th %^‡^	*n*	Highest quintile	*n*	Highest quintile	*n*	Highest quintile	*n*	Lowest quintile	*n*	Highest quintile	*n*	Highest quintile	*n*	Highest quintile
Overall	3555	26.5	13.8	3456	57.2	2521	0.13	1754	182	1752	42	1751	128	625	109	626	103

*Girls*																	
Non-Hispanic Black	516	23.4	13.0	506	55.6	365	0.10	268	182	268	44	268	127	100	112	100	87
Hispanic	694	33.9	14.6	670	57.9	502	0.20	346	181	344	41	344	127	113	102	113	110
Non-Hispanic White	530	24.0	10.8	513	57.0	341	0.11	238	188	238	40	238	135	90	103	90	105
*Boys*																	
Non-Hispanic Black	555	18.0	12.4	538	55.5	391	0.08	272	183	272	46	272	123	120	108	120	85
Hispanic	684	18.9	19.0	660	59.0	516	0.16	368	179	369	42	368	124	119	105	120	112
Non-Hispanic White	576	18.9	11.8	569	57.2	406	0.10	262	181	261	41	261	130	83	112	83	118

*Centers for Disease Control and Prevention standardized cut-point values for age and sex

^†^Cut-point for defining overweight

^‡^Cut-point for defining obesity

**Table 2:** Demographic characteristics of 3,644 3-to-6-year-olds in the National Health and Nutrition Examination Survey, 1999–2008.

	*N*	Weighted *N*	Percent	95% CI
*Gender*				
Boys	1,865	7,399,751	50.9	48.6–53.2
Girls	1,779	7,135,318	49.1	46.8–51.4
*Age (years) *				
3	922	3,493,552	24.0	22.1–26.0
4	976	3,701,859	25.5	23.5–27.4
5	887	3,581,816	24.6	22.7–26.6
6	859	3,757,841	25.9	23.9–27.8
*Ethnicity*				
Non-Hispanic White	1,138	9,000,820	61.9	58.2–65.7
Hispanic	1,416	3,285,391	22.6	19.5–25.7
Non-Hispanic Black	1,090	2,248,857	15.5	13.0–17.9

Total	3,644	14,535,068		

**Table 3:** Sample, estimated national characteristics, and range of values for measurements in 3,644 3-to-6-year-olds in the National Health and Nutrition Examination Survey, 1999–2008.

	*N*	Weighted *N*	Mean	95% CI
Total sample	3,644	14,535,068	4.52	4.48–4.57

*Anthropometrics *				
BMI (kg/m^2^)	3,555	14,165,622	16.30	16.20–16.39
Waist Circumference (cm)	3,456	13,795,833	53.86	53.56–54.17
*CVD risk factor*				
C-reactive protein (mg/dL)	2,521	9,679,388	0.16	0.13–0.19
Total cholesterol (mg/dL)	1,754	6,895,867	161.88	160.07–163.68
HDL cholesterol (mg/dL)	1,752	6,885,787	51.95	51.18–52.72
Non-HDL cholesterol (mg/dL)	1,751	6,884,098	20.8	17.9–23.6
LDL cholesterol (mg/dL)	625	5,807,247	92.75	90.58–94.92
Triglycerides (mg/dL)	626	5,811,466	84.87	78.37–91.37

Prenatal, infant, and social risk factor	*N*	Weighted *N*	Percent	95% CI

Breastfed	3,628	14,485,571	64.8	61.7–67.9
Solid food <6 months	3,594	14,340,199	42.0	39.4–44.5
Smoking while pregnant	3,626	14,459,865	17.4	14.9–19.8
Low birth weight	3,343	13,299,081	9.7	8.1–11.3
Poverty income ratio <1	3,378	13,649,782	25.3	23.0–27.6
Poverty income ratio <2	3,378	13,649,782	51.6	48.2–55.0

CVD risk factor	*N*	Weighted *N*	Percent	95% CI

BMI ≥85 %ile for age, sex	3,555	14,165,622	24.5	22.6–26.3
BMI ≥95 %ile for age, sex	3,555	14,165,622	12.7	11.3–14.1
Waist circumference^1^	3,456	13,795,833	20.7	18.8–22.6
C-reactive protein^1^	2,521	9,679,388	21.0	18.9–23.0
Total cholesterol^1^	1,754	6,895,867	21.0	18.6–23.5
HDL cholesterol^2^	1,752	6,885,787	18.3	15.7–20.8
LDL cholesterol^1^	625	5,807,247	23.3	19.2–27.4
Triglyceride^1^	626	5,811,466	21.8	16.9–26.8

^1^Highest quintile

^2^Lowest quintile

**Table 4:** Elevated cardiovascular disease risk factors among US 3-to-6-year-olds by ethnicity and infant nutrition practices, National Health and Nutrition Examination Survey, 1999–2008.

	Hispanic		Non-Hispanic Black		Non-Hispanic White	
Perinatal factor	Odds ratio (95% CI)	*P*-value	Odds ratio (95% CI)	*P*-value	Odds ratio (95% CI)	*P* value
*Breastfed* (Y/N)						
BMI ≥ 85th %ile for age, sex	1.14 (0.82–1.57)	0.43	0.84 (0.58–1.22)	0.37	0.61 (0.42–0.87)	**0.01**
BMI ≥ 95th %ile for age, sex	0.96 (0.65–1.41)	0.83	0.82 (0.53–1.28)	0.39	0.43 (0.27–0.69)	**<0.001**
Waist circumference^1^	1.33 (0.91–1.93)	0.14	0.71 (0.44–1.14)	0.16	0.58 (0.37–0.92)	**0.02**
C-reactive protein^1^	0.78 (0.53–1.16)	0.22	1.24 (0.84–1.82)	0.28	0.76 (0.52–1.13)	0.18
Total cholesterol^1^	1.45 (0.83–2.55)	0.19	0.62 (0.33–1.15)	0.13	0.92 (0.49–1.70)	0.78
HDL cholesterol^2^	0.98 (0.63–1.52)	0.93	0.87 (0.41–1.85)	0.73	0.48 (0.23–1.04)	0.06
Non-HDL cholesterol^1^	1.23 (0.70–2.14)	0.48	0.56 (0.32–0.98)	**0.04**	1.10 (0.60–2.02)	0.76
LDL cholesterol^1^	1.90 (1.01–3.55)	**0.05**	0.74 (0.30–1.86)	0.53	1.89 (0.52–6.83)	0.33
Triglycerides^1^	2.01 (0.71–5.71)	0.19	1.24 (0.56–2.74)	0.59	1.40 (0.47–4.19)	0.54
*Solids < 6 months* (Y/N)						
BMI ≥ 85th %ile for age, sex	1.06 (0.77–1.46)	0.72	1.13 (0.81–1.56)	0.47	1.17 (0.82–1.68)	0.38
BMI ≥ 95th %ile for age, sex	0.85 (0.61–1.20)	0.36	1.03 (0.68–1.56)	0.90	1.28 (0.82–1.99)	0.28
Waist circumference^1^	0.90 (0.60–1.35)	0.61	1.05 (0.75–1.45)	0.79	1.38 (0.96–1.98)	0.09
C-reactive protein^1^	0.79 (0.50–1.23)	0.29	0.80 (0.57–1.12)	0.20	1.50 (0.99–2.26)	0.06
Total cholesterol^1^	1.08 (0.59–1.98)	0.80	1.07 (0.58–1.99)	0.83	1.39 (0.76–2.54)	0.29
HDL cholesterol^2^	0.79 (0.45–1.37)	0.41	0.93 (0.52–1.69)	0.82	0.67 (0.38–1.16)	0.15
Non-HDL cholesterol^1^	0.85 (0.48–1.50)	0.57	1.60 (0.91–2.81)	0.10	1.34 (0.80–2.23)	0.27
LDL cholesterol^1^	0.33 (0.12–0.94)	**0.04**	1.47 (0.74–2.90)	0.27	0.87 (0.36–2.08)	0.75
Triglycerides^1^	0.71 (0.26–1.92)	0.50	0.80 (0.30–2.09)	0.64	0.90 (0.30–2.69)	0.85

^1^Highest quintile

^2^Lowest quintile

**Table 5:** Elevated cardiovascular disease risk factors among US 3-to-6-year-olds by ethnic group and maternal smoking during pregnancy, National Health and Nutrition Examination Survey, 1999–2008.

	Hispanic		Non-Hispanic Black		Non-Hispanic White	
Perinatal Factor	Odds Ratio (95% CI)	*P*-value	Odds ratio (95% CI)	*P*-value	Odds ratio (95% CI)	*P*-value
*Maternal Smoking* (Y/N)						
BMI ≥ 85th %ile for age, sex	1.49 (0.82–2.73)	0.19	0.99 (0.53–1.85)	0.98	1.79 (1.13–2.83)	**0.01**
BMI ≥ 95th %ile for age, sex	1.29 (0.62–2.68)	0.49	1.35 (0.58–3.14)	0.48	2.22 (1.30–3.78)	**<0.01**
Waist circumference^1^	1.06 (0.56–1.98)	0.87	1.18 (0.65–2.12)	0.59	1.40 (0.83–2.36)	0.20
C-reactive protein^1^	0.85 (0.38–1.90)	0.69	1.23 (0.56–2.75)	0.61	1.63 (1.05–2.51)	**0.03**
Total cholesterol^1^	0.61 (0.18–2.02)	0.42	0.62 (0.19–2.06)	0.43	0.73 (0.39–1.40)	0.35
HDL cholesterol^2^	0.61 (0.19–1.93)	0.40	1.11 (0.56–2.18)	0.77	1.29 (0.58–2.86)	0.53
Non-HDL cholesterol^1^	0.58 (0.18–1.82)	0.35	0.67 (0.19–2.41)	0.54	0.79 (0.46–1.36)	0.40
LDL cholesterol^1^	0.71 (0.16–3.26)	0.66	0.65 (0.14–2.99)	0.58	0.24 (0.06–0.90)	**0.03**
Triglycerides^1^	0.71 (0.15–3.31)	0.66	0.58 (0.15–2.25)	0.43	1.39 (0.34–5.69)	0.64

^1^Highest Quintile

^2^Lowest Quintile

**Table 6:** Elevated cardiovascular disease risk factors among US 3-to-6-year-olds if low birth weight, by ethnic group, National Health and Nutrition Examination Survey, 1999–2008.

	Hispanic		Non-Hispanic Black		Non-Hispanic White	
Perinatal Factor	Odds Ratio (95% CI)	*P*-value	Odds Ratio (95% CI)	*P*-value	Odds Ratio (95% CI)	*P*-value
*Low birth weight* (Y/N)						
BMI ≥ 85th %ile for age, sex	0.82 (0.47–1.44)	0.49	0.88 (0.44–1.37)	0.38	0.33 (0.14–0.76)	**0.01**
BMI ≥ 95th %ile for age, sex	0.99 (0.54–1.83)	0.97	1.01 (0.46–2.23)	0.98	0.37 (0.11–1.22)	0.10
Waist circumference^1^	0.89 (0.43–1.86)	0.89	1.03 (0.56–1.88)	0.93	0.34 (0.16–0.74)	**<0.01**
C-reactive protein^1^	1.74 (0.94–3.22)	0.08	1.37 (0.81–2.29)	0.24	0.51 (0.17–1.50)	0.22
Total cholesterol^1^	1.58 (0.66–3.83)	0.31	0.64 (0.28–1.44)	0.28	1.51 (0.54–4.21)	0.43
HDL cholesterol^2^	1.37 (0.53–3.54)	0.52	1.14 (0.53–2.47)	0.74	1.28 (0.32–5.08)	0.72
Non-HDL cholesterol^1^	1.88 (0.75–4.68)	0.18	0.55 (0.25–1.25)	0.16	0.81 (0.26–2.53)	0.72
LDL cholesterol^1^	1.58 (0.40–6.23)	0.51	0.87 (0.19–3.96)	0.85	0.39 (0.02–6.71)	0.52
Triglycerides^1^	0.98 (0.20–4.71)	0.98	0.99 (0.40–2.43)	0.99	1.37 (0.22–8.49)	0.74

^1^Highest quintile

^2^Lowest quintile
